# Association between prediabetes and periodontitis: a meta-analysis of observational studies with multivariate analysis

**DOI:** 10.4317/medoral.26961

**Published:** 2025-02-15

**Authors:** Yin Zhou, Fei Sun, Qiuyan Zhu

**Affiliations:** 1 Department of Periodontics, Shaoxing Stomatological Hospital, Shaoxing, Zhejiang Province, China

## Abstract

**Background:**

Growing evidence suggests that prediabetes may increase the risk of periodontitis, though the extent of this association remains unclear. To provide a clearer understanding, this meta-analysis focused on observational studies that utilized multivariate analyses to adjust for key confounding factors.

**Material and Methods:**

A comprehensive search of PubMed, Embase, and Web of Science was conducted to identify observational studies assessing the relationship between prediabetes and periodontitis. Only studies that utilized multivariate analyses were included to minimize confounding bias. The quality of the studies was evaluated with the Newcastle-Ottawa Scale (NOS). Odds ratios (ORs) and 95% confidence intervals (CIs) were calculated using a random-effects model, with heterogeneity assessed by the I² statistic.

**Results:**

Ten observational studies with 38,727 participants were included. Overall, individuals with prediabetes had a significantly higher risk of periodontitis compared to normoglycemic individuals (OR: 1.27, 95% CI: 1.09 to 1.48, *p* < 0.001) with moderate heterogeneity (I² = 53%). Subgroup analyses revealed a stronger association in studies where the proportion of men was < 45% compared to those ≥ 45% (OR: 1.75 vs. 1.15, *p* for subgroup difference = 0.01). Studies with lower quality (NOS score = 7) showed a stronger association compared to higher-quality studies (NOS score = 8 or 9, *p* for subgroup difference = 0.003).

Conclusion: This meta-analysis found that prediabetes may be independently associated with an increased risk of periodontitis. Further research is needed to explore the mechanisms underlying this association and potential sex-specific effects.

** Key words:**Prediabetes, periodontitis, risk factor, observational studies, meta-analysis.

## Introduction

Periodontitis is a chronic inflammatory disease affecting the supporting structures of the teeth, including the gingiva, periodontal ligament, and alveolar bone (1). If it is left untreated, periodontitis can lead to tooth loss and has been increasingly associated with systemic conditions such as cardiovascular diseases, diabetes, and respiratory diseases (2). According to global epidemiological data, periodontitis affects approximately 45-50% of the adult population worldwide, with severe cases occurring in 10-15% of individuals (3). The adverse effects of periodontitis extend beyond oral health, as chronic inflammation and bacterial burden can trigger or exacerbate systemic conditions (4). Given its prevalence and systemic implications, early prevention and treatment of periodontitis are crucial in reducing its overall burden (5).

Known risk factors for periodontitis include poor oral hygiene, smoking, advanced age, obesity, and genetic predisposition (2). Additionally, certain systemic conditions, such as diabetes, are well-established risk factors for periodontitis (6). Diabetes exacerbates the inflammatory response in periodontal tissues, leading to increased susceptibility to periodontitis and more severe disease progression. Consequently, the bidirectional relationship between diabetes and periodontitis has been extensively studied and is well-acknowledged in both dental and medical fields (6). Early prevention of periodontitis, particularly in individuals at higher risk, plays a key role in mitigating long-term oral and systemic health complications.

Prediabetes, an intermediate state between normoglycemia and diabetes, is characterized by elevated blood glucose levels that do not yet meet the diagnostic criteria for diabetes (7). It is typically diagnosed through impaired fasting glucose (IFG), impaired glucose tolerance (IGT), or mildly elevated hemoglobin A1c (HbA1c) levels (8). Prediabetes is a critical stage in the progression to type 2 diabetes and poses a significant public health concern, affecting an estimated 374 million people worldwide (9). While the link between diabetes and periodontitis is well-documented, the relationship between prediabetes and periodontitis remains less clear, despite the increasing global prevalence of prediabetes (10). Understanding whether prediabetes contributes to the development of periodontitis is essential for establishing targeted prevention strategies.

Current evidence regarding the association between prediabetes and periodontitis is mixed. Many observational studies suggest a higher risk of periodontitis in individuals with prediabetes, but most of these findings are derived from univariate analyses that do not fully adjust for confounding factors such as age, sex, body mass index (BMI), and smoking etc. (11-13). In contrast, the results of studies utilizing multivariate analyses to account for these variables have been inconsistent, with some studies reporting a significant association (14-17) while others do not (18-23). This inconsistency highlights the need for a comprehensive meta-analysis that focuses solely on studies employing multivariate analysis to clarify the true relationship between prediabetes and periodontitis. In view of these knowledge gaps, the aim of this meta-analysis is to rigorously evaluate the association between prediabetes and periodontitis by synthesizing data from observational studies that adjust for confounding factors through multivariate analysis.

## Material and Methods

The authors adhered to PRISMA 2020 guidelines (24) and the Cochrane Handbook for Systematic Reviews and Meta-analyses (25) in conducting this meta-analysis, covering study design, data collection, statistical analysis, and result interpretation. The protocol of the manuscript has been registered in PROSPERO with the identifier CRD42024597452.

- Literature search

To identify studies pertinent to this meta-analysis, we queried PubMed, Embase, and Web of Science with an extensive array of search terms, which included: 1) "prediabetes" OR "pre-diabetes" OR "prediabetic" OR "pre-diabetic" OR "prediabetic state" OR "borderline diabetes" OR "impaired fasting glucose" OR "impaired glucose tolerance" OR "IFG" OR "IGT"; and 2) "periodontal" OR "periodontitis" OR "oral health". The search was limited to research involving human subjects and included only studies published as full-text articles in English within peer-reviewed journals. The detailed search strategy for each database is provided in Supplement 1. Additionally, we manually reviewed the references of relevant original and review articles to identify further pertinent studies. The literature was assessed from the inception of the databases up to August 20, 2024.

- Inclusion and exclusion criteria

The inclusion criteria for potential studies were defined according to the PICOS framework:

P (Population): General population such as community derived population without specific diagnosis of diseases.

I (Exposure): Participants with prediabetes, which was diagnosed according to the criteria used in the primary studies involving IFG, IGT, and mildly elevated HbA1c.

C (Comparison): Participants with normoglycemia.

O (Outcome): Incidence or prevalence of periodontitis, which was compared between those with prediabetes and normoglycemia and analyzed in a multivariate analysis at least adjusting age and sex. The diagnosis of periodontitis was also consistent with the methods and criteria used in the primary studies. These typically included well-defined criteria from the 2017 World Workshop on the Classification of Periodontal Diseases (26), the Centers for Disease Control and Prevention (CDC) and the American Academy of Periodontology (AAP) criteria (27), the European Workshop in Periodontology (28), or self-defined criteria using periodontal examinations for bleeding on probing (BOP), probing depth (PD), clinical attachment level (CAL), radiographic evidence of bone loss, or the modified Community Periodontal Index (CPI).

S (Study Design): Observational studies, including cohort, case-control, and cross-sectional studies.

Exclusion criteria included reviews, editorials, meta-analyses, preclinical studies, studies including patients with specific diagnosis of diseases, studies that did not evaluate PreD as exposure, or studies that did not report the outcome related to the prevalence or the incidence of periodontitis. To avoid the potential influence of confounding factors, studies that only reported outcome data of univariate analysis were excluded. In cases of overlapping populations, the study with the largest sample size was selected for inclusion in the meta-analysis.

- Study quality evaluation and data extraction

The literature search, study identification, quality assessment, and data extraction were conducted independently by two authors, with any disagreements resolved by consulting the Correspondence. Study quality was evaluated using the Newcastle-Ottawa Scale (NOS) (29), which assesses selection, control of confounders, and outcome measurement and analysis, with scores ranging from 1 to 9, where 9 signifies the highest quality. Data collected for analysis included study details (author, year, country, and design), participant characteristics (source, sample size, age, and sex), the criteria for the diagnosis of prediabetes and number of subjects with prediabetes, follow-up periods for longitudinal studies, criteria or methods used for the diagnosis of periodontitis, numbers of patients with periodontitis, and variables adjusted when analyzing the association between prediabetes and periodontitis.

- Statistical analyses

The association between prediabetes and periodontitis was analyzed using odds ratios (ORs) and 95% confidence intervals (CI), comparing between subjects with prediabetes and normoglycemia. OR values and their standard errors were calculated from 95% CIs or *p-value*s and logarithmically transformed for variance stabilization. To assess heterogeneity, we used the Cochrane Q test and I² statistics (25), with I² > 50% indicating considerable heterogeneity. A random-effects model was applied to integrate the results, accounting for study variability (25). Sensitivity analyses were conducted by excluding individual studies to test the robustness of the findings. Predefined subgroup analyses were performed to explore the effects of factors such as study design, average age, sex, definition of prediabetes, methods or criteria used for the diagnosis of periodontitis, and NOS scores. Subgroups were defined using the median values of continuous variables. Publication bias was evaluated using funnel plots and visual inspection for asymmetry, supplemented by Egger’s regression test (30). Analyses were performed using RevMan (Version 5.1; Cochrane Collaboration, Oxford, UK) and Stata software (version 17.0; Stata Corporation, College Station, TX).

## Results

- Study inclusion

**Figure 1:**
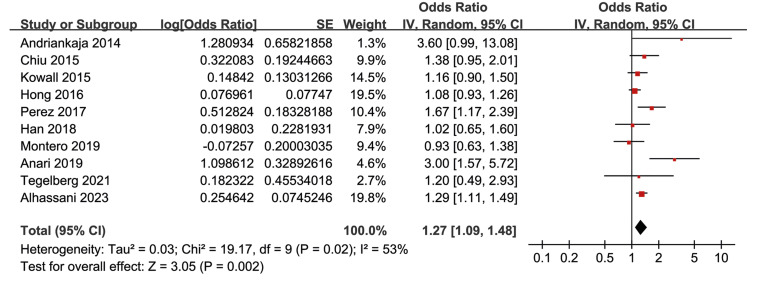
Forest plots representing the meta-analysis of the association between prediabetes and periodontitis in general population.

The study inclusion process is illustrated in Fig. 1. Initially, 821 potentially relevant records were identified from the three databases, with 339 excluded due to duplication. A subsequent screening of titles and abstracts led to the exclusion of 449 studies, primarily because they did not align with the objective of the meta-analysis. The full texts of the remaining 33 records were reviewed by two independent authors, resulting in the exclusion of 23 studies for reasons detailed in Supplement 2. Ultimately, ten observational studies were deemed appropriate for the quantitative analysis (14-23).

- Overview of study characteristics

Table 1 presents the summarized characteristics of the included studies. Overall, one prospective cohort study (18), one retrospective cohort study (23), seven cross-sectional studies (14,15,17,19-22), and another case-control study (16) were included. These studies were reported from 2014 to 2023, and conducted in Puerto Rico, Taiwan, Germany, Korea, Spain, Iran, Finland, and the United States.

Community populations were included in nine studies (14,15,17-23), and the other case-control study included people with prediabetes and healthy control (16). Overall, 38727 participants were included in this meta-analysis, with the mean ages from 31.0 to 51.3 years, and the proportion of men varying from 21.2 to 60.6%. Prediabetes was defined as IFG in four studies (18,20,22,23), IFG and/or IGT in two studies (14,19), mildly elevated HbA1c three studies (16,17,21), and IFG, IGT, or mildly elevated HbA1c in another study (15). The follow-up durations for the two cohort studies were 5 and 12 years, respectively. The diagnosis of periodontitis was according to the CDC/AAP definition in two studies (15,17), via the CPI score ≥ 3 in four studies (18,20,21,23), with the EWP criteria in one study (19), and using other criteria such as BOP (14), CAL (22), and diagnosis in medical records (16) in another three studies. Overall, 12611 (32.6%) of the included participants were diagnosed as periodontitis. Multivariate analyses were performed in all of the included studies, and the potential confounding factors such as age, sex, BMI, smoking, alcohol drinking etc. were adjusted to a varying degree. The NOS scores of the included cohort studies (Supplement 3) and cross-sectional or case-control studies (Supplement 4) were seven to nine, suggesting an overall good study quality.

- Results of meta-analysis and sensitivity analysis

Overall, the pooled results of ten studies indicated that people with prediabetes, as compared to people with normoglycemia, were associated with a higher risk of periodontitis (OR: 1.27, 95% CI: 1.09 to 1.48, *p* < 0.001) with moderate heterogeneity (I2 = 53%; Fig. 1). Sensitivity analysis by excluding one dataset at a time did not significantly change the results (OR: 1.21 to 1.33, *p* all < 0.05).

- Results of the subgroup analyses

**Figure 2:**
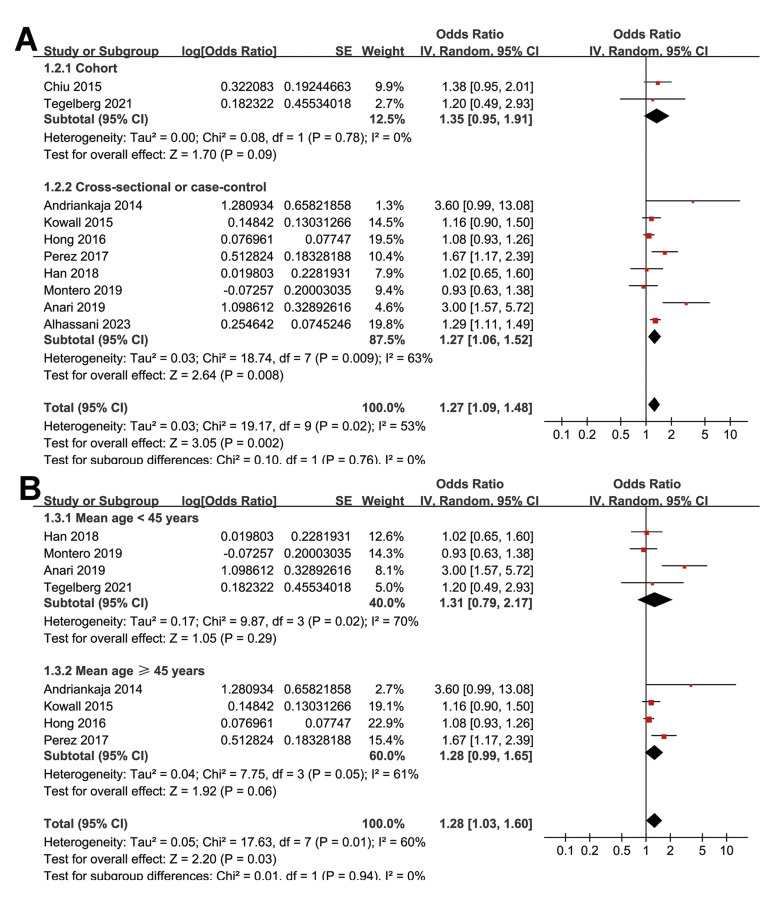
Forest plots representing the subgroup analyses of the association between prediabetes and periodontitis; A, subgroup analysis according to study design; and B, subgroup analysis according to the mean age of the participants.

**Figure 3:**
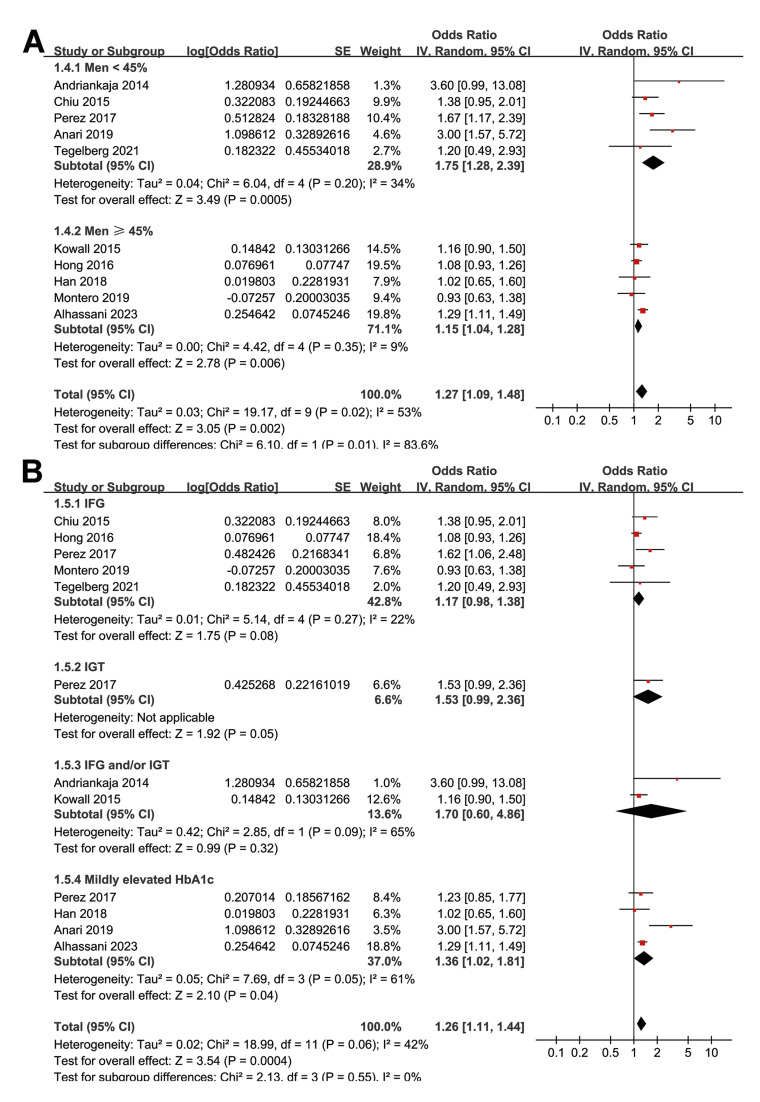
Forest plots representing the subgroup analyses of the association between prediabetes and periodontitis; A, subgroup analysis according to the proportion of men; and B, subgroup analysis according to the definition of prediabetes.

**Figure 4:**
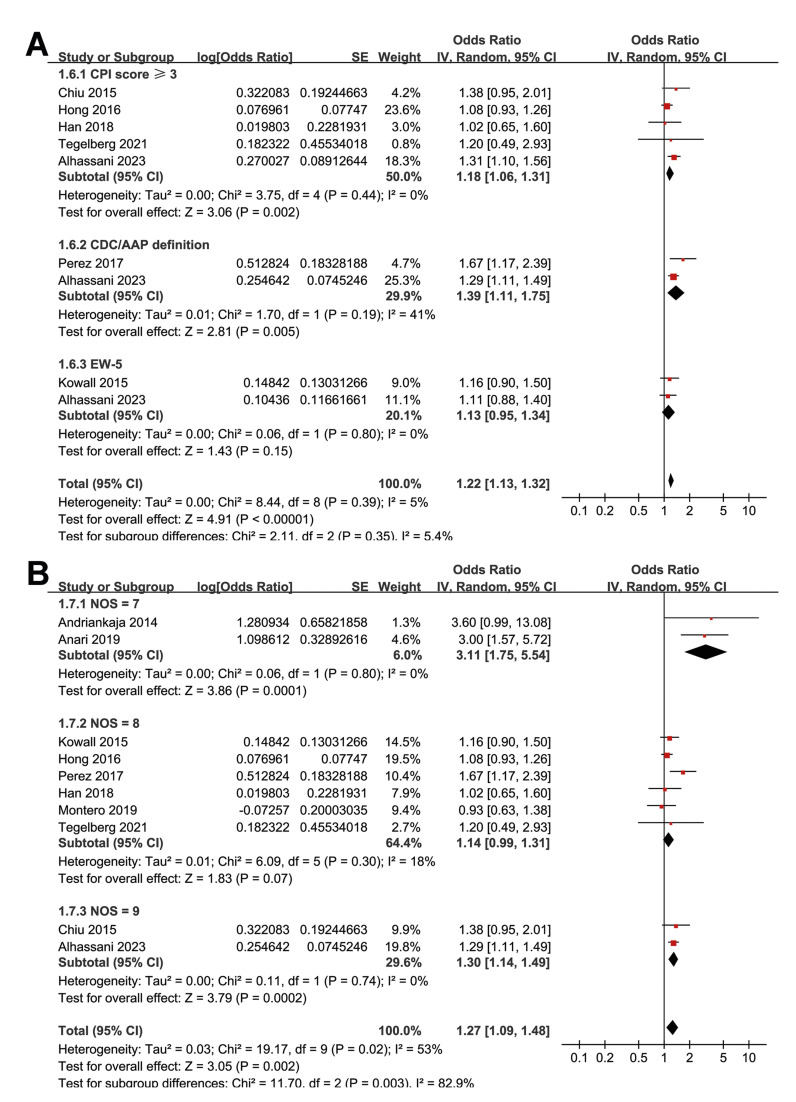
Forest plots representing the subgroup analyses of the association between prediabetes and periodontitis; A, subgroup analysis according to the criteria for the diagnosis of periodontitis; and B, subgroup analysis according to the NOS scores of the included studies.

Subgroup analyses indicated that the association between prediabetes and periodontitis was not significantly different between cohort and cross-sectional/case control studies (*p* for subgroup difference = 0.76; Fig. 2) or between studies with the mean age of the participants < or ≥ 45 years (*p* for subgroup difference = 0.94; Fig. 2). Interestingly, a stronger association between prediabetes and periodontitis was observed in studies with the proportion of men < 45% as compared to those with men ≥ 45% (OR: 1.75 versus 1.15, *p* for subgroup difference = 0.01; Fig. 3). Further subgroup analyses did not suggest that the association between prediabetes and periodontitis could be significantly affected by the definition of prediabetes (*p* for subgroup difference = 0.55; Fig. 3) or the criteria for the diagnosis of periodontitis (*p* for subgroup difference = 0.35; Fig. 4). Finally, a stronger association between prediabetes and periodontitis was observed in studies with the NOS score of 7 as compared to those with 8 or 9 (OR: 3.11 versus 1.14 and 1.30, *p* for subgroup difference = 0.003; Fig. 4).

- Publication bias

Upon visual inspection, the funnel plots for meta-analysis of the association between prediabetes and periodontitis appear symmetrical, indicating a low likelihood of publication bias (Supplement 5). Additionally, Egger’s regression test results (*p* = 0.42) also support this conclusion by suggesting a low risk of publication bias.

## Discussion

This meta-analysis synthesized evidence from observational studies with multivariate adjustments to assess the association between prediabetes and periodontitis. Our findings suggest that prediabetes is significantly associated with an increased risk of periodontitis, even after adjusting for potential confounding factors such as age, smoking, and BMI. The pooled results underscore the importance of recognizing prediabetes as a potential risk factor for periodontitis, further reinforcing the need for early intervention and prevention strategies in populations with impaired glucose metabolism.

Several plausible biological mechanisms could explain the association between prediabetes and periodontitis. Prediabetes is characterized by chronic hyperglycemia and insulin resistance, both of which have detrimental effects on periodontal tissues (31). Elevated blood glucose levels promote the formation of advanced glycation end-products (AGEs), which accumulate in tissues and contribute to increased oxidative stress and inflammation (32). These AGEs bind to specific receptors (RAGEs) on various cell types, including macrophages and endothelial cells, triggering the release of pro-inflammatory cytokines such as tumor necrosis factor-α (TNF-α) and interleukin-6 (IL-6) (33). This inflammatory cascade exacerbates periodontal destruction by impairing tissue repair and enhancing collagen degradation, ultimately leading to alveolar bone loss and tooth mobility (34). Moreover, insulin resistance in prediabetes may further impair the immune response, diminishing the body's ability to control bacterial infections in the periodontal tissues, thereby increasing susceptibility to periodontitis (35). Studies are still warranted in the future to determine the key molecular mechanisms underlying the association between prediabetes and periodontitis.

In our subgroup analysis, a possible influence of participant sex and study quality on the association between prediabetes and periodontitis was observed. Studies with a higher percentage of male participants showed a slightly stronger association between prediabetes and periodontitis. This finding may be explained by gender-specific differences in metabolic and immune responses. Men may exhibit more pronounced insulin resistance and inflammation compared to women, which may exacerbate the periodontal inflammatory response (36). Additionally, behavioral factors, such as poorer oral hygiene and higher rates of smoking among men, may contribute to the stronger association observed in this subgroup (37). However, the gender-based difference was modest, and more research is needed to fully elucidate the potential biological and behavioral reasons behind this disparity. The subgroup analysis also revealed that studies of higher methodological quality (i.e., studies with higher NOS scores) tended to report weaker associations between prediabetes and periodontitis. This may reflect a bias in lower-quality studies, where confounding factors such as socioeconomic status and other comorbidities might not have been adequately controlled. Higher-quality studies with more rigorous multivariate adjustments may provide more accurate estimates, which could explain the attenuated effect size in this subgroup. However, despite the reduced effect size in these high-quality studies, the overall association between prediabetes and periodontitis remained significant, indicating that the relationship persists even after accounting for study quality.

One of the strengths of this meta-analysis is its focus on studies that used multivariate analysis, which minimizes the influence of confounding variables and provides a more accurate representation of the true relationship between prediabetes and periodontitis. Additionally, our analysis includes a large number of participants from diverse populations, enhancing the generalizability of the findings. However, several limitations should be acknowledged. First, despite our efforts to include only studies with multivariate adjustments, the included studies still varied in their adjustment for confounders. Not all studies adjusted for the same variables, which may have introduced some residual confounding. Second, the observational nature of the included studies prevents us from establishing a causal relationship between prediabetes and periodontitis. While the biological plausibility of this association is supported by mechanistic studies, further longitudinal research is needed to determine whether prediabetes directly contributes to the development of periodontitis. Third, the heterogeneity observed in some subgroup analyses suggests that other unmeasured factors, such as differences in diagnostic criteria for periodontitis or variations in glucose thresholds for prediabetes, may have influenced the results. Finally, similar to diabetes, the association between prediabetes and periodontitis is also likely to be bidirectional. Studies are warranted for further evaluation.

In this meta-analysis, we observed that the included studies used varying criteria to diagnose both prediabetes and periodontitis, which may contribute to the heterogeneity of our findings. Prediabetes was diagnosed using different thresholds of fasting glucose, HbA1c, or glucose tolerance tests, which can impact the identified prevalence and progression of prediabetes. Similarly, periodontitis diagnostic methods varied, with some studies relying on CAL or pd measurements, while others used radiographic evidence or composite diagnostic criteria. This inconsistency could influence the observed association, as less precise diagnostic criteria might lead to under- or overestimation of disease burden. Future studies would benefit from standardized diagnostic protocols to improve comparability across research and strengthen the robustness of pooled analyses.

From a clinical perspective, the findings of this meta-analysis have important implications for both dental and medical practice. Recognizing prediabetes as a risk factor for periodontitis highlights the need for interdisciplinary care between dentists, endocrinologists, and primary care physicians. Individuals with prediabetes should be considered at high risk for periodontal disease, warranting closer monitoring of oral health and early preventive interventions. Regular dental check-ups and periodontal screenings should be recommended for patients with prediabetes to mitigate the risk of disease progression. Additionally, public health efforts should focus on raising awareness about the potential oral health implications of prediabetes and promoting lifestyle modifications that address both systemic and periodontal health, such as improved glycemic control and smoking cessation (38). Future research should aim to address several key gaps. Longitudinal studies are needed to establish a temporal relationship between prediabetes and periodontitis and to assess whether controlling prediabetes can reduce the risk or severity of periodontal disease. Moreover, additional studies that explore the role of gender, socioeconomic status, and other demographic factors in modulating this relationship would provide a more nuanced understanding of at-risk populations. Further investigations into the underlying molecular mechanisms, particularly the role of AGEs, insulin resistance, and inflammatory pathways, could also uncover new therapeutic targets for preventing or treating periodontitis in individuals with prediabetes (39).

## Conclusions

In conclusion, this meta-analysis suggests that prediabetes is associated with an increased risk of periodontitis, even after adjusting for confounding factors. The findings emphasize the importance of early identification and management of prediabetes, not only to prevent the progression to diabetes but also to reduce the risk of periodontal complications. Given the global rise in prediabetes prevalence, incorporating periodontal health assessments into the routine care of individuals with prediabetes could have significant public health benefits. Further research is needed to confirm these findings and to explore potential interventions that target both systemic and oral health in this population.

## Figures and Tables

**Table 1:** Characteristics of the included studies.

Study	Location	Design	Characteristics of population	No. of participants	Mean age (years)	Men (%)	Definition of PreD	No. of subjects with PreD	Follow-up duration for cohort studies	Diagnosis of periodontitis	No. of subjects with periodontitis	Variables adjusted
Andriankaja 2014	Puerto Rico	CS	Community adults aged 40-65 years	94	51.3	30.9	IFG and/or IGT	17	NA	Periodontal examination (% BOP ≥ median)	51	Age, sex, smoking status, alcohol consumption, WC, and number of missing teeth
Chiu 2015	Taiwan	PC	Community population aged 35-44 years	4387	NR	21.2	IFG	297	5	CPI score ≥ 3	1247	Age, sex, education, smoking, alcohol drinking, WC, TG, HDL-C, HTN, fruit intake, and physical activity
Kowall 2015	Germany	CS	Community adults aged 20-82 years	2721	46.9	49.4	IFG and/or IGT	576	NA	EWP-5 criteria	607	Age, sex, BMI, education, smoking, alcohol consumption, TC, HDL-C and TG
Hong 2016	Korea	CS	Community population aged 19 years or older	9977	45.1	50.7	IFG	2126	NA	CPI score ≥ 3	2728	Age, sex, smoking, alcohol drinking, SES, WC, HDL-C, TG, and hypertension
Perez 2017	Puerto Rico	CS	Community adults aged 40-65 years	1191	49.9	27.2	IFG and/or IGT and/or HbA1c (5.7-6.4%)	683	NA	CDC/AAP definition	784	Age, sex, education, smoking status, physical activity, alcohol consumption, WC, HDL-C, and plaque index
Han 2018	Korea	CS	Community adults aged 19 years or older	8341	40.6	47.4	HbA1c (6.0-6.4%)	NR	NA	CPI score ≥ 3	1968	Age, sex, smoking, drinking, education, income, BMI, number of natural teeth, frequency of tooth brushing per day, use of secondary oral products and duration of sleep
Montero 2019	Spain	CS	Community employed population	4241	38.7	60.6	IFG	373	NA	CAL ≥ 6	1609	Age, sex, occupation, education, smoking, BMI, WC, TG, TC, and SBP/DBP
Anari 2019	Iran	CC	PreD people and healthy controls	302	42.2	39.1	HbA1c (5.7-6.4%)	151	NA	Clinically diagnosed as evidenced by medical chart	58	Age, sex, having denture, and smoking status
Tegelberg 2021	Finland	RC	Community people aged 31 years	639	31	41.2	IFG	203	12	CPI score ≥ 3	270	Sex, BMI, education, number of teeth with plaque and number of tooth sites
Alhassani 2023	USA	CS	Community adults aged 30 years or older	6834	NR	53.5	HbA1c (5.7-6.4%)	2050	NA	CDC/AAP definition	3289	Age, sex, race, smoking, BMI, SES, education, and physical activity

PreD, prediabetes; CS, cross-sectional; PC, prospective cohort; RC, retrospective cohort; CC, case-control; NR, not reported; NA, not available; DM, diabetes mellitus; IFG, impaired fasting glucose; IGT, impaired glucose tolerance; CDC/AAP, Centers for Disease Control and Prevention/American Academy of Periodontology; BOP, bleeding on probing; CPI, Community Periodontal Index; EWP, European Workshop in Periodontology; CAL, clinical attachment loss; WC, waist circumference; TG, triglyceride; HDL-C, high-density lipoprotein cholesterol; HTN, hypertension; BMI, body mass index; TC, total cholesterol; SES, socioeconomic status; SBP, systolic blood pressure; DBP, diastolic blood pressure.
